# Nuclear Magnetic Resonance Analysis Seeking for Metabolic Markers of Hypertension in Human Serum

**DOI:** 10.3390/molecules30102145

**Published:** 2025-05-13

**Authors:** Adriana Sousa, Nádia Oliveira, Ricardo Conde, Elisabete Morais, Ana Paula Amaral, Nieves Embade, Oscar Millet, Ignacio Verde

**Affiliations:** 1 Health Sciences Research Centre (CICS-UBI), University of Beira Interior (UBI), 6200-506 Covilhã, Portugal; amssousa@fcsaude.ubi.pt (A.S.); nadiaoliveira@fcsaude.ubi.pt (N.O.); ralves@cicbiogune.es (R.C.); elisabete.morais@fcsaude.ubi.pt (E.M.); anapaula@fcsaude.ubi.pt (A.P.A.); 2 RISE-Health, Faculty of Health Sciences, University of Beira Interior (UBI), Av. Infante D. Henrique, 6200-506 Covilhã, Portugal; 3 Center for Cooperative Research in Biosciences (CIC-BioGUNE), Bizkaia Science and Technology Park, 48160 Derio, Spain; nembade@cicbiogune.es (N.E.); omillet@cicbiogune.es (O.M.)

**Keywords:** hypertension, biomarkers, aging, NMR, metabolomics

## Abstract

Hypertension is a highly prevalent medical condition that occurs when blood pressure is too high, which greatly increases the risk of developing other cardiovascular diseases and is generally associated with higher rates of morbidity and mortality. Due to the silent/asymptomatic nature of hypertension, although the methods currently available to diagnose it are easy, they generally do not allow for an early diagnosis and an efficient prognosis to avoid irreversible damage in the medium or long term. In fact, an early diagnosis of hypertension would be crucial to decrease hypertension-associated mortality. Since metabolomics using NMR can provide a global measurement of various serum metabolites, it is very suitable for detecting novel biomarkers. We therefore analyzed serum metabolomic profiles among normotensive and hypertensive elderly individuals by NMR and identified new potential biomarkers for hypertension and associated diseases. We found higher levels of acetate, formate, and glycerol, and lower levels of glutamine, glycine, and sarcosine in individuals with hypertension. Therefore, these metabolites could be used for early diagnosis of hypertension to avoid comorbidities derived from hypertension and associated mortality.

## 1. Introduction

Hypertension is a chronic condition that occurs when the blood pressure is too high. Hypertension strongly raises the risk of developing other cardiovascular diseases and is associated with higher morbidity and mortality rates [1,2]. In fact, hypertension is associated with more than 10 million deaths every year worldwide, mainly linked to cardiovascular diseases, stroke, and chronic kidney disease [1,2]. Hypertension has a prevalence of 33% in people 30–79 years old, and its prevalence increases with age progression [1,2]. According to the latest report from World Health Organization (WHO), 46% of the hypertensive people 30–79 years old are not diagnosed and are completely unaware of their condition [1]. Therefore, achieving an early and accurate diagnosis is crucial for early treatment, avoiding irreversible damage and decreasing associated mortality.

Frequent measurement of the blood pressure (BP) is the current available method for identifying and diagnosing hypertension [3,4]. However, BP measurements are not often performed as recommended until patients start presenting symptoms associated with other cardiovascular problems. Also, BP fluctuates depending on the patient’s emotional state, consumptions of products such as coffee or tobacco, or adequate rest period during measurement. Thus, an early diagnosis of hypertension, based also on a greater number of parameters, distinct from the increase in blood pressure and the appearance of other associated pathologies, would be crucial to reduce associated complications and mortality. Furthermore, the detection of these associated metabolites can be useful to understand the molecular basis of hypertension. Also, several pathophysiological changes can occur before the BP is highly increased. WHO stated that high BP levels increase the risk of death even with levels in the range of 115–130 mmHg of systolic BP [1,3,4].

Metabolomic analysis has been used in the last years to identify novel disease biomarkers, including cardiovascular diseases, using technical approaches such as nuclear magnetic resonance (NMR) and mass spectrometry, which allowed the measurement of a vast number of metabolites in different samples to analyze changes related to these diseases [2,4,5,6,7]. In this sense, we used NMR, which is a rapid and accurate analytical technique, with a high throughput and high reproducibility [8,9].

Previous studies have highlighted a connection between intestinal dysbiosis and hypertension [10,11,12]. Several authors have reported significantly different concentrations of metabolites produced by gut microbiota, such as formate, hippurate, 4-hydroxyhippurate, and lyxose, in blood and urine of hypertensive participants when compared with healthy normotensive individuals [13,14,15].

The pathogenesis of hypertension has also been associated with inflammation and oxidative stress [16]. In this sense, some studies have reported changes in metabolites associated with these states and hypertension, such as the amino acids glycine and serine, and some phosphatidylcholines, which have anti-inflammatory and antioxidant properties [2,17,18,19]. Hao et al. also found differences in threonine and phenylalanine between normotensive and hypertensive participants [15]. Hypertension has also been associated with glucose metabolism and insulin resistance [20], and some authors have found differences in several metabolic markers associated with glycolysis, such as glucose, glycerol, and lactate [2,5,18,21]. These preceding studies were performed using NMR or mass spectrometry in blood and urine samples from the individuals. The findings were not consensual among the different studies, none of them was performed specifically in aged individuals (65 years or older), and some studies used targeted metabolomic approaches, limiting the results to few metabolites.

Therefore, we aimed to analyze the serum metabolomic profiles between normotensive (control) and hypertensive participants by using untargeted NMR to identify novel biomarkers for hypertension in a population with a high prevalence of hypertension.

## 2. Results

### 2.1. Sociodemographic and Clinical Data

Differences in sociodemographic characteristics and health status of the individuals could influence the metabolite levels of the different study groups. Thus, we summarized in Table 1 and Table 2 several parameters that will be considered later in the analysis. Regarding sex and hypertension diagnosis, although it seems that there is a higher percentage of women in the HTall group compared with NT group, these results did not reach statistical significance. The average age of the HTall group was significantly higher than that of the NT group. (Table 1). However, we did not find significant differences between NT and HTo in these parameters. Interestingly, there were no differences in the average values of systolic and diastolic blood pressures, either between NT and HTall or between NT and HTo, suggesting that monitoring and applied treatment in the LTCF can effectively control the elevated blood pressure levels (Table 1). Mean BMI values of the NT, HTall, and HTo groups corresponded to the overweight range (25–29.9 kg/m^2^), and there were no significant differences between groups (Table 1).

Regarding serum biochemistry data, the average values of glucose, triglycerides, total cholesterol, LDL cholesterol, and HDL cholesterol obtained in the NT, HTo, and HTall groups correspond to the levels considered normal for the non-hypertensive population. We did not find significant differences in the mean levels of glucose, triglycerides, and HDL-cholesterol between samples of the NT and HTall groups. In contrast, individuals from the HTall group had lower levels of total cholesterol than people from the NT group (Table 1). In this sense, it seems that, although in the HTall group, the proportion of people with dyslipidemia (47.0%) and diabetes (31.3%) was high (Table 2), the participants seemed to be properly monitored and treated for these conditions. No significant differences were found between NT and HTo in the biochemistry parameters.

Concerning cognitive tests, the ACE-R, MMSE, and GDS, the average scores were not significantly different between NT and HTall nor between NT and HTo (Table 1).

Regarding cardiovascular and metabolic comorbidities, almost half of the participants with hypertension had a diagnosis of dyslipidemia (44.8%). Also, we found high prevalence of diabetes (34.2%) and cardiac diseases, such as chronic heart failure (23.5%), and arrhythmia (23.0%), in the HTall group. With lower prevalence, we also found that several participants with hypertension had diagnosis of angina (10.2%), peripheric vascular disease (8.8%), valvular pathologies (4.8%), and history of acute myocardial infarction (3.7%). As previously described, in the NT group, all the participants with cardiovascular and metabolic conditions were excluded. Also, the HTo group is constituted of participants that had a hypertension diagnosis but no other CVMD.

Table 2 shows the percentages of use of different classes of drugs used by individuals in the NT and HTall groups. As could be expected, there is a significant correlation between the occurrence of hypertension and the increased proportion of some drugs used to treat hypertension, such as angiotensin-receptor antagonists (ARA), calcium channel blockers (CCB), and diuretics (Table 2). Also, there is a significant correlation between the use of anticoagulants and HTall. Other drug classes are more widely used in the HTall group, such as angiotensin-converting enzyme inhibitors (ACEi) or beta-adrenergic antagonists (BAA), but the differences are not significant. As expected, we observed a significantly increasing correlation in the use of anticoagulants from NT to HTall group (Table 2).

Table 2 also compares the percentages of use of different classes of drugs between the NT and HTo groups. There is a significant correlation between the hypertension diagnosis and the increased proportion of some drugs used to treat hypertension, such as ACEi, ARA, and diuretics.

In general, the proportion of medications used to treat metabolic disorders is also higher in the HTall group, but no significant correlations were observed. No significant correlations were observed in the use of bronchodilators or the treatment of CNS diseases between the NT and HTall or between NT and HTo (Table 3).

### 2.2. Univariate Analysis of Metabolites

**Figure 1:**
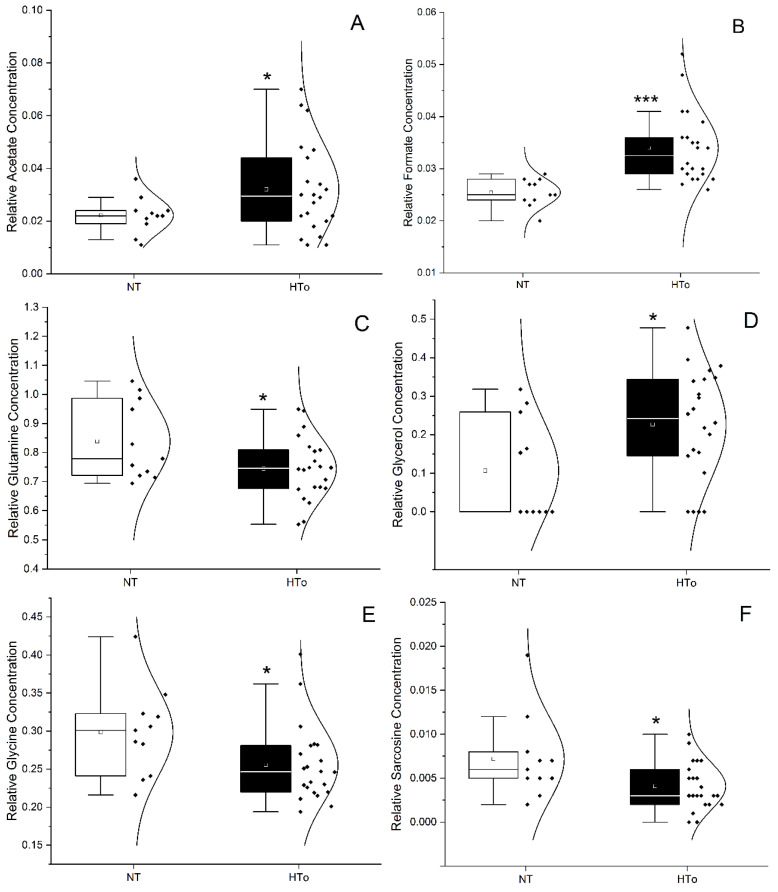
Violin plots representing the variation of metabolite concentration between NT group vs. HTo group for acetate (**A**), formate (**B**), glutamine (**C**), glycerol (**D**), glycine (**E**) and sarcosine (**F**). *t*-test or Mann–Whitney test (* *p* < 0.05, *** *p* < 0.001 vs. NT group).

**Figure 2:**
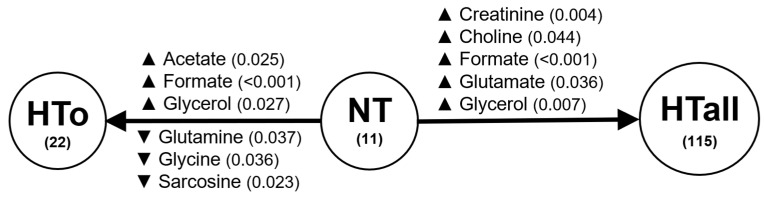
Summary of changes in metabolite levels observed between NT group and HTo (**left**) and HTall (**right**). The numbers below the group names indicate the number (n). ▲/▼ = increase/decrease in metabolite levels. Statistical significances were analyzed using Student’s *t*-test, *p* is indicated between brackets after the metabolite names.

To study the changes in metabolomic profiles influenced by hypertension, we first analyzed the serum metabolite concentration differences between the NT (control) and the HTo group, constituted of individuals only diagnosed with hypertension and without other cardiovascular diseases. The levels of acetate, formate, and glycerol were increased in the HTo group when compared with the NT group. Other metabolite levels, such as glutamine, glycine, and sarcosine, were significantly decreased in the HTo group when compared with the NT group (Figure 1 and Figure 2 and Table S1).

Formate and glycerol also increased from NT to HTall, but no significant differences were found in acetate, glutamine, glycine, or sarcosine when comparing these groups. Regarding other metabolites, glutamate and creatinine levels were significantly increased in HTall when compared with the NT group (Figure 2, Table S2).

**Figure 3:**
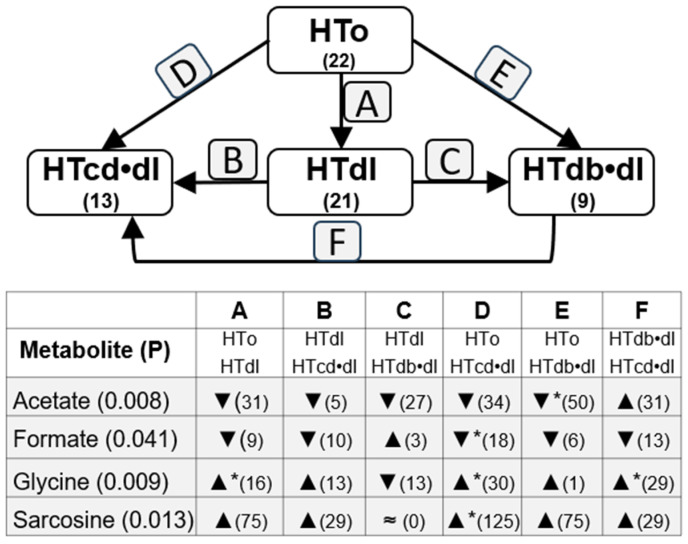
Differences in levels of metabolites observed between HTo and subgroups with individuals diagnosed with dyslipidemia (HTdl), dyslipidemia plus diabetes (HTdb•dl), and dyslipidemia plus one cardiac disease (HTcd•dl). The numbers below the group names indicate the number (n). The bottom box indicates metabolites; ▲—increase or ▼—decrease (percentage of change). Statistical significance was analyzed using the Kruskal–Wallis test (KWT) or One-way ANOVA test (OWAT) depending on the homogeneity of variance. Differences among subgroups were analyzed by using the post hoc Tukey test or all pairwise nonparametric method (* *p* < 0.05). “≈” means similar (variation is lower that 0.5%).

**Figure 4:**
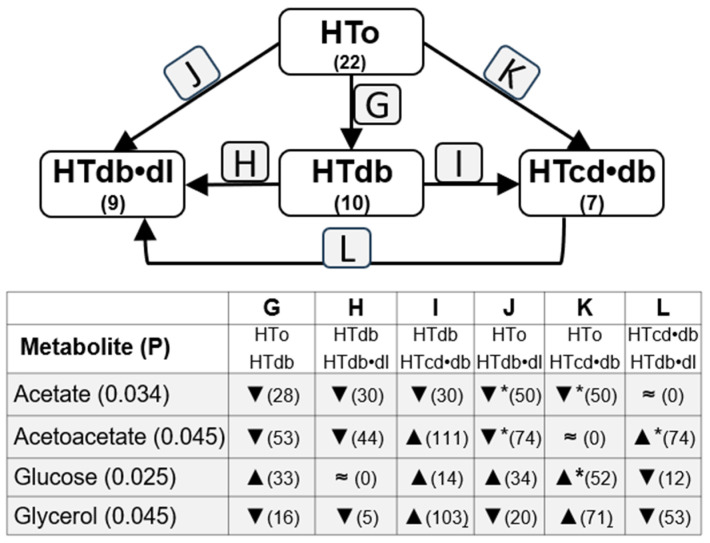
Differences in levels of metabolites observed between HTo and subgroups with individuals diagnosed with diabetes (HTdb), dyslipidemia plus diabetes (HTdb•dl), and diabetes plus one cardiac disease (HTcd•db). The bottom box indicates metabolites; ▲—increase or ▼—decrease (percentage change). Statistical significance was analyzed using the Kruskal–Wallis test (KWT) or One-way ANOVA test (OWAT) depending on the homogeneity of variance. Differences among subgroups were analyzed by using the post hoc Tukey test or all pairwise nonparametric method (* *p* < 0.05). “≈” means similar (variation is lower that 0.5%).

**Figure 5:**
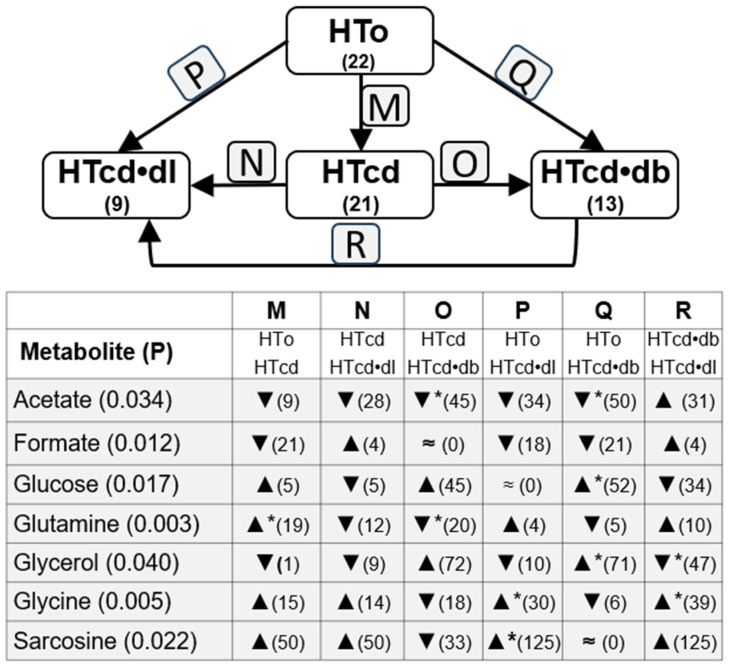
Significant differences in levels of metabolites observed between HTo and subgroups of individuals diagnosed with cardiac disease (HTcd), dyslipidemia plus cardiac disease (HTcd•dl), and diabetes plus cardiac disease (HTcd•db). The numbers below the group names indicate the number (n). The bottom box indicates metabolites; ▲—increase, ▼—decrease (percentage change). Statistical significance was analyzed using the Kruskal–Wallis test (KWT) or One-way ANOVA test (OWAT) depending on the homogeneity of variance. Differences among subgroups were analyzed by using the post hoc Tukey test or all pairwise nonparametric method (* *p* < 0.05). “≈” means similar (variation is lower that 0.5%).

To understand the influence of other cardiovascular diseases in the metabolite concentrations among the NT and HTall groups, we also compared these metabolite concentrations between the subgroups related to different comorbidities, such as diabetes, dyslipidemia, and cardiac diseases. Thus, Figure 3 shows changes related to dyslipidemia, including three subgroups: the HTdl subgroup, with hypertensive individuals diagnosed with diabetes but without any other cardiac diseases; the HTdb•dl subgroup, with hypertensive individuals diagnosed with dyslipidemia and diabetes but without any other cardiac disease diagnosed; and the HTcd•dl subgroup, with hypertensive individuals diagnosed with dyslipidemia, and only one cardiac disease (heart failure, arrhythmia, or history of acute myocardial infarction), without other cardiac diseases diagnosed. Analogous analysis was performed with changes related to diabetes (Figure 4) and cardiac diseases (Figure 5).

Acetate serum levels are different among HTo and HTdl, HTcd•dl, and HTdb•dl subgroups, and the post hoc test indicated the main significant decrease in its levels in the HTdb•dl subgroup when compared with HTo. Also, a lower level in HTdl (*p* = 0.056) and HTcd•dl (*p* = 0.078) was found when compared with HTo, although these differences did not reach statistical significance (Figure 3, Table S3). Thus, acetate increases with hypertension, but diabetes and dyslipidemia can induce a decrease in serum acetate levels. Also, changes were observed among subgroups HTo vs. HTdb, HTdb•dl, and HTcd•db (Figure 4, Table S4), once again suggesting that diabetes can decrease acetate serum levels. The post hoc test highlighted mainly the differences among HTo and HTdb•dl or HTcd•db, although a great difference was also observed with between HTdb and HTdb•dl and HTcd•db, still suggesting a great contribution of dyslipidemia or cardiac diseases. The analysis of subgroups related to cardiac diseases (comparison among HTo and HTcd, HTcd•dl, and HTcd•db) confirmed that diabetes is a main influencer on serum acetate decrease (Figure 5, Table S5). These data could explain the absence of significant changes in acetate levels between NT and HTall.

Formate serum levels showed significant differences among the HTo, HTdl, HTcd•dl, and HTdb•dl subgroups, and the post hoc test indicated a main significant decrease between the HTo and HTcd•dl subgroups (Figure 3, Table S3). The analysis of formate levels of subgroups related to cardiac diseases (comparison among HTo, HTcd, HTcd•dl, and HTcd•db) also showed differences among these subgroups (Figure 5, Table S5). The post hoc test showed relevant but not significant differences among HTo and HTcd (*p* = 0.069, 21% of decrease), HTcd•dl (*p* = 0.051, decrease of 18%), or HTcd•db (*p* = 0.077, decrease of 21%). Thus, formate also increases with hypertension, but dyslipidemia and cardiac diseases seem to induce a decrease in its levels: these data could explain the absence of significant changes in formate levels between NT and HTall.

Concerning glycerol, we found significant differences among HTo, HTcd, HTcd•dl, and HTcd•db, and the post hoc test indicated a significant increase from HTo to the HTcd•db subgroup, and a significant decrease from HTcd•db to HTcd•dl (Figure 5, Table S5). Thus, glycerol increases with hypertension and further increases when hypertension coexists with diabetes and a cardiac disease.

Glutamine serum levels were different among HTo, HTcd, HTcd•dl, and HTcd•db subgroups, mainly because its level increased from HTo to HTcd (Figure 5, Table S5). However, the concentration of glutamine is significantly lower in the HTcd•db subgroup than in the HTcd subgroup and is relevantly lower in HTcd•dl when compared with HTcd (*p* = 0.074, decrease of 12%). Thus, this metabolite is significantly decreased in individuals that have only hypertension, but the concomitant presence of cardiac disease increases glutamine. However, the increase caused by the association of cardiac disease seems to be mitigated if there exists concomitance of cardiac disease and diabetes, or cardiac disease and dyslipidemia. These opposite effects can justify the absence of differences between NT and HTall.

Glycine levels were significantly different among the HTo, HTdl, HTcd•dl, and HTdb•dl subgroups. The post hoc test showed a significant increase between HTo and HTdl or HTcd•dl, and an increase from HTdb•dl to HTcd•dl (Figure 3, Table S3). Significant differences were also found among the HTo, HTcd, HTcd•dl, and HTcd•db subgroups. The post hoc test still indicated an increase from HTo to HTcd•dl and, additionally, from HTcd•db to HTcd•dl (Figure 5, Table S5). Thus, hypertension decreases the glycine levels, but dyslipidemia and cardiac diseases positively influence glycine levels, dyslipidemia being more influent in the increase in glycine. These counterbalanced effects can justify the absence of differences between NT and HTall.

Sarcosine levels were different among the HTo, HTdl, HTcd•dl, and HTdb•dl subgroups. The post hoc test showed main differences from HTo to HTcd•dl (Figure 3, Table S3). The comparison of sarcosine levels among HTo, HTcd, HTcd•dl, and HTcd•db showed differences, and once again, the main differences were found between HTo and HTcd•dl (Figure 5, Table S5). It is worthy to note that NMR technology does not detect some physiological levels of this metabolite (0.006–0.014 mM) because the limit of detection is 0.01 mM (Table S6). Nevertheless, sarcosine levels are decreased when individuals have hypertension, but the levels are increased when hypertension coexists with dyslipidemia and one cardiac disease.

Finally, glucose levels were increased when individuals were diagnosed with diabetes, significantly from HTo to HTcd•db, and although a ~33% increase was found between HTo and HTdb, this difference was not statistically significant (Figure 4, Table S4). Glucose levels were not different among NT and HTo nor between NT and HTall. Acetoacetate levels were different among the HTo, HTdb, HTcd•db, and HTdb•dl subgroups, and the post hoc test displayed significant differences increasing from HTo to HTdb•dl and decreasing from HTcd•db and HTdb•dl. Acetoacetate levels were not different among NT and HTo nor between NT and HTall. No differences in creatinine or glutamate were found among the subgroups analyzed in this section.

In summary, dyslipidemia seems to have an influence in many metabolites, such as acetate (mitigating the increase occurring in HTo), and in glutamine, glycine, and sarcosine (mitigating the decrease occurring in HTo). On the other hand, diabetes only seemed to influence acetate levels (mitigating the increase in HTo). Lastly, cardiac diseases seemed to influence formate and glutamine, mitigating the increase and decrease, respectively, occurring in HTo.

**Figure 6:**
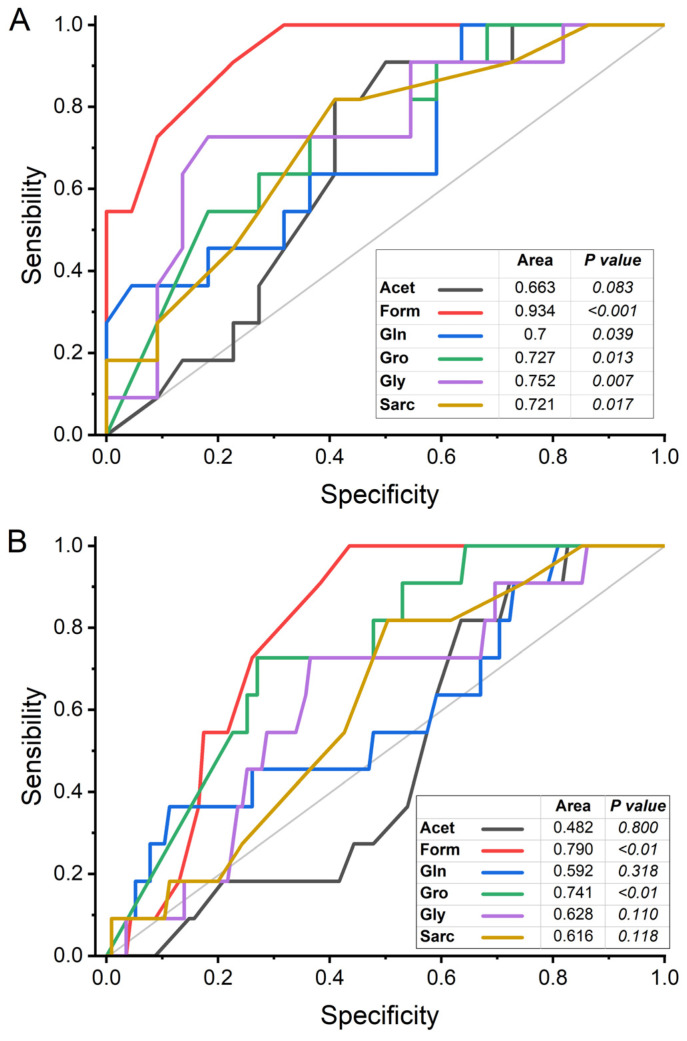
AUROC analysis to discriminate isolated hypertension (**A**) and hypertension with CVMD (**B**) for the serum levels of the following metabolites: acetate (Acet), formate (Form), glutamine (Gln), glycerol (Gro), glycine (Gly), and sarcosine (Sarc). The diagonal grey line is typical of ROC courves and divides the ROC space (new as random classifier). Points above the diagonal represent good classification results (better than random); points below the line represent bad results (worse than random).

The discriminatory power of hypertension for the six metabolites that showed significant differences between the study groups was estimated from the analysis of the area under the receiver operating characteristic curve (AUROC; sensitivity/specificity), using the concentrations obtained in the univariate analysis. When this analysis was performed with the NT and HTo groups, formate, glutamine, glycerol, glycine, and sarcosine showed an AUROC equal to or greater than 0.7, with significant *p* values, indicating good discriminatory power for discriminating hypertension when other CVMDs do not coexist with hypertension. In these conditions, acetate had an AUROC less than 0.7, with a non-significant *p* value (Figure 6A). Differently, when AUROC analysis was performed with the NT and HTall groups, only formate and glycerol showed an AUROC value greater than 0.7 and significant *p* values, suggesting that these two metabolites can be discriminative of hypertension even in the presence of CVMD. On the contrary, the AUROC of acetate, glutamine, glycine, and sarcosine showed AUROC values under 0.63 and no significant *p* values, suggesting a poor discriminant power for hypertension when coexisting with other CVMD (Figure 6B).

### 2.3. Analysis of Possible Influence of Drug Treatments

As the changes in metabolites detected between subgroups could be influenced by the drugs used to treat cardiovascular diseases, and the use of some of these drugs varies between these subgroups, we analyzed whether there were differences in the levels of these metabolites between the individuals in the subgroups that used these drugs and individuals in the subgroups that were not medicated for these conditions.

Thus, we studied if ACEi, ARA, BAA, CCB, diuretics, and anticoagulants influenced the metabolites’ levels, because these are the drug classes that have been differently and significantly used when we compared NT with HTo or with HTall (Table 2). We only analyzed the effect of these drugs on acetate, acetoacetate, formate, glucose, glutamine, glycerol, glycine, or sarcosine levels, because these are the metabolites that showed statistical differences among the groups or subgroups studied.

We did not have a convenient number of individuals in the HTall group with monotherapy with each one of the drug classes to compare the metabolite levels with individuals without ACEi, ARA, BAA, CCB, diuretics, and anticoagulant therapy. Thus, we compared the metabolite levels between sets of individuals using therapies with one or more of these therapeutic classes and individuals without these therapies (control set). In this sense, Table 3 shows the means and the ratio of changes (%) of acetate, acetoacetate, formate, and glucose between individuals of the control set, and Table 4 shows the percent changes of glutamine, glycerol, glycine, and sarcosine.

The statistical comparison of the mean values (STT) to analyze the influence of drugs did not reveal significant differences in most of the metabolite levels due to the use of these therapies when compared with the control set of individuals without these therapies. The singular significant difference was only found in sarcosine between the control set and the set of individuals with ACEi + AnCoa + CCB + DIU. Despite this, we observed some changes of more than 20% between these sets for several metabolites that will be commented on in the next paragraphs.

As mentioned before, acetate serum levels significantly increased with hypertension when comparing the NT and HTo groups (Table 2). Table 3 showed that, even if the differences are not significant, individuals treated just with ACEi have higher levels of acetate than individuals without the therapies analyzed. On the other hand, individuals treated only with ACEi + AnCoa (*p* = 0.096, STT) or just with ARA (*p* = 0.06, STT) had lower levels of acetate. Other therapies did not elicit changes higher than 20%. The increase in acetate by ACEi did not seem to have a big influence on the significant higher level of acetate (45%) observed in HTo because only 9% of individuals in the HTo group were being treated with this therapy. Treatment with ARA or ACEI + AnCoA had the opposite influence observed with the existence of hypertension.

We did not find changes between the NT, HTo, and HTall groups concerning acetoacetate serum levels (Table 2), but we found a decrease when comparing the HTo group with the HTdb or HTdb•dl subgroups (Figure 4). Here, we observe that individuals treated with ACEi + AnCoa + DIU had higher levels of this metabolite (Table 3), even if the differences were not significant (*p* = 0.400) and, thus, this therapy could not be a positive influence on the decrease in acetoacetate in the HTdb or HTdb•dl subgroups. On the other hand, individuals treated only with ACEi, with ACEi + AnCoa, ACEi + AnCoa + CCB + DIU with ARA + DIU, with ARA + AnCoa + CCB, with DIU, DIU + AnCoa, or DIU + AnCoa + BAA, showed (39 total) levels of acetate decreased more than 20% when compared with the control set, mainly DIU with a decrease of 65.1% (*p* = 0.083), even if these differences are not statistically significant. It is worth indicating that more than 45% of individuals were taking these types of therapies; meanwhile, for the HTdb and HTdb•dl subgroups, 20% and 44% of individuals, respectively, were taking these types of therapies. Thus, we assume that the differences concerning acetoacetate levels observed with the HTdb (decrease of ~53%) or HTdb•dl (decrease of ~74%) subgroups are not related to these therapies.

Concerning formate, its serum levels were increased in HTo and HTall when compared with the NT group (Table 2 and Figure 2). Now, we observed that none of the therapeutic conditions analyzed increased their levels. However, several combinations induced decreases of more than 20% in formate levels (Table 3), such as ACEi + AnCoa, ARA + AnCoa + CCB, and DIU + AnCoa + BAA. Thus, these therapies can only have influence veiling the probable condition of this metabolite as a biomarker of hypertension.

Glucose did not show changes related to hypertension among the hypertension subgroups studied and was increased in the subgroups related to diabetes (HTdb, HTdb•dl, and HTcd•db) when compared with HTo (Table 2 and Figure 3). Concerning therapies, glucose levels increased more than 30% in individuals treated with ARA + AnCoa + CCB, a therapy that was observed in only 23% of the individuals of the subgroups related to diabetes.

Glutamine levels were higher in HTo when compared with NT (Table 2 and Figure 2). None of the therapies analyzed significantly influenced glutamine levels and the changes induced were mainly below ±10% (Table 4). Only the therapy Diu + AnCoa + BAA decreased glutamine levels 10.1% (*p* = 0.053, STT), but there were no individuals in the HTo group with this therapy.

Glycerol levels were increased in the HTo and HTall groups when compared with the NT group (Table 2 and Figure 2). This metabolite was not significantly influenced by the analyzed therapies, although it increased by more than 30% by ARA + DIU, DIU + AnCoa + BAA, ARA + AnCoa + DIU + CCB therapies when compared with people without the therapies (Table 4). Only 14% of the individuals in HTo and only ~21% in HTall were using this therapy, which suggests that there is not a big effect on the differences found in glycerol related to hypertension. On the other hand, DIU, ARA + AnCoa + CCB, and DIU + AnCoa + BAA elicited a decrease in glycerol by more than 30%, but clearly, in this case, these therapies were not promoting the increase in glycerol related to hypertension.

Glycine serum concentration decreased in HTo when compared with the NT group (Table 2 and Figure 2). None of the analyzed therapeutics showed significant influence in glycine levels, and none of them changed the glycine levels by more than 20% (Table 4). Thus, these therapeutics did not influence the findings related to the decrease in glycine related to hypertension.

Concerning sarcosine, its serum concentration decreased in HTo (~43%) when compared with the NT group (Table 2). Also, this is the metabolite with the lowest mean concentration that than can be related to hypertension. In this scope, the data showed that several therapies analyzed positively influenced serum concentration with a highest and significant increase observed in individuals treated with ACEi + AnCoa + CCB + DIU (145%). Also, other therapies increased sarcosine levels by more than 60%, such as ARA (82.5%), ARA + AnCoa + CCB (137.5%), and DIU + AnCoa (70%), but these increases were not statistically significant. Only around 4.5% of the individuals in HTo were treated with ACEi + AnCoa + CCB + DIU and none with ARA + AnCoa + CCB, ARA, or DIU + AnCoa. Thus, these data suggest that the decrease in sarcosine in HTo is related to hypertension and not with the used therapies, because the therapies analyzed have increasing influence in sarcosine levels.

## 3. Discussion

The identification of clinical biomarkers is essential to improve the diagnosis and prognosis of hypertension and cardiovascular diseases. Although some studies have looked at biomarkers for cardiovascular disease, these findings are still controversial and inconclusive. These studies mainly analyzed groups of cardiovascular disease patients compared with healthy individuals in different types of populations. The use of 1H-NMR in plasma from individuals from LTCF allowed the analysis of a population with similar living conditions, in a specific age group, and in which there are individuals with and without cardiovascular diseases. Thus, through univariate statistical analysis, we quantified potential metabolic biomarkers related to hypertension and associated diseases.

The mean age of individuals with hypertension was higher than that of NT individuals, which agrees with the fact that the prevalence of hypertension increases with age [1]. This may be due to changes in arterial and arteriolar stiffness that occur with aging, associated with structural changes, increased in the peripheral vascular resistance in small vessels, and changes in mechanisms such as baroreceptor sensitivity, responsiveness to the sympathetic nervous system, renal ionic homeostasis, and renin-aldosterone system functioning [22]. Environmental factors can also play an important role, related to poor diet, obesity, physical inactivity, alcohol and tobacco consumption, and exposure to pollutants [1]. We observed a higher proportion of women diagnosed with hypertension compared to men, but these results did not reach statistical significance and are most likely related to the higher proportion of women in the LTCF population in which our study was conducted. On the other hand, although at younger ages, the prevalence of hypertension is higher in men, after female menopause, this trend is reversed and women have a higher prevalence [23]. According to WHO, the global prevalence of hypertension in people aged 30 to 79 years is higher in men than in women [1].

We did not find differences in MMSE, ACE-R, and GDS scores between the NT and HTall or HTo groups. The comorbidities found in the HTall group (dyslipidemia, diabetes, and other cardiovascular diseases) correspond to those expected in hypertensive individuals according to what was reported by other authors [24,25]. The data also show that there were no differences in mean systolic and diastolic BP between participants in the NT and HTall groups, probably because most individuals in the LTCF studied who were diagnosed with hypertension were diagnosed and were closely monitored and receiving treatment to control hypertension. In this broader sense, WHO indicated that of the 2.7 million Portuguese citizens diagnosed with hypertension aged between 30 and 79 years, 69% were correctly diagnosed (vs. 54% globally) and 63% were being treated (vs. 42% globally) [1]. Also, a study published in 2019 concluded that 69.8% of Portuguese people diagnosed with hypertension were diagnosed, 69.4% are treated, and 71.3% have their blood pressure under control [26]. However, other studies in other geographic areas such as China [4,27,28,29] and India [3] have shown significant differences between normotensive and hypertensive individuals, probably because these countries have a lower rate of treated hypertensive individuals and lower magnitudes of effectively controlled hypertensive individuals (below 17%) [1].

Regarding BMI and obesity, we found no significant differences between the NT and hypertension groups. Several studies, conducted with adults (18–89 years old), also found no significant differences [4,15,18,29,30]. However, some studies have reported an association between untreated hypertension and BMI levels, although this is attenuated in adequately treated individuals. Thus, one study (individuals aged 35–80 years) found a significant increase in SBP and DBP per unit of BMI in participants without antihypertensive treatment, which is substantially weaker in treated individuals [31]. The results of these studies suggest the importance of effective BP control in preventing cases of overweight and obesity. These results support the fact that we might not have found a significant difference in BMI between both groups because most participants were taking the antihypertensive medication correctly and the SBP and DBP were being properly controlled.

Regarding clinical biochemistry parameters, glucose, triglycerides, and LDL cholesterol, there are no significant differences between the NT and HTall groups. Other authors have also reported the absence of significant differences in these levels between normotensive and hypertensive individuals [15,27]. Zong et al. found similar levels of triglycerides and LDL cholesterol, but increased glucose, total cholesterol, and triglycerides [29]. Ke et al. found similar levels of HDL cholesterol, but increased glucose, triglycerides, and LDL cholesterol. Different studies have reported other differences such as increases in serum levels of triglycerides [3,30], glucose [30], total cholesterol [32], and LDL cholesterol [3], or a decrease in HDL cholesterol [3] in hypertensive patients.

The comparison between NT and HTo showed that hypertensive individuals have higher levels of acetate, formate, and glycerol. Acetate is one of the most common and the shortest of the fatty acids. Short fatty acids are produced by the gut microbiota in the large intestine, as fermentation products from ingested food that was not completely absorbed in the small intestine [33]. This is of relevance as hypertension diagnosis has started to be associated with intestinal dysbiosis [10,11]. In our study, participants from the HTo group presented higher serum levels of acetate; however, this increase was mitigated in the presence of concomitant dyslipidemia and/or diabetes diagnosis. Moreover, the AUROC analysis showed that the discriminant power of acetate for hypertension is lower than the other metabolites found in this study and did not reach statistical significance. Regarding the association between acetate and hypertension, another study, performed in 20 human participants without pharmacologic treatment for hypertension, supplemented the participants with sustained high levels of both acetate and butyrate. After this supplementation, the hypertensive participants had a significant reduction of 6.1 mmHg in 24 h SBP (vs. participants that were given a placebo) [34]. A previous study, performed with mice, described that high-fiber diet (which increased the abundance of acetate-producing bacteria) combined with acetate supplementation (vs. mineralocorticoid-excess diet) led to decreased intestinal dysbiosis, and decreased SBP, DBP, cardiac fibrosis, and left ventricular hypertrophy [35]. Thus, we hypothesize that the increase in acetate in the hypertensive participants found in our study might be due to intestinal dysbiosis occurring in hypertension, and possibly as a compensatory mechanism to protect against hypertension. The medication of individuals with ACEi, ARA, diuretics, BAA, CCB, anticoagulants, or its combinations, did not significantly modify the acetate serum levels, and most of them elicited no significant decrease in acetate, whereas acetate levels are increased with hypertension.

Formate and its conjugate formic acid (the simplest carboxylic acid) are essential endogenous metabolites at the heart of the one-carbon pathway, present in most living organisms [36]. Formate can be produced in a folate dependent and independent way, and it can be produced as a byproduct of fermentation of dietary fiber by the gut microbiome [13,36]. Because of its association with folate, plasma and urine levels are generally increased during folate and vitamin B12 deficiencies [36]. In our study, we found that participants diagnosed with hypertension had higher formate serum levels. However, when analyzing participants with hypertension and associated comorbidities (HTall group), we found that the concomitant presence of CVMD mitigated this increase. Nevertheless, the AUROC analysis showed a good discriminant power of formate for hypertension, independently of the coexistence of other CVMDs. Other authors found elevated formate levels relates to metabolic syndrome with diabetes, obesity, dyslipidemia, and hypertension [37]. Other researchers studied formate levels in urine, and Holmes et al. found a significant negative association between the mean 24 h urinary formate excretion levels and both SBP and DBP. These authors also found a positive correlation between the urinary formate levels and the energy intake, as well as with 24 h urinary Na+ and K+ levels [13]. Because of these results, mainly the association between formate levels and Na+, and the importance of NaCl in the control of BP, the authors suggested that formate might have an unrecognized role in BP regulation. Besides hypertension, plasma formate levels also seem to be associated with stroke risk, as shown by another study performed by our team [38]. The medication of individuals with ACEi, ARA, diuretics, BAA, CCB, anticoagulants, or its combinations decreases, although not significantly, the formate serum levels, in the opposite way than hypertension. Thus, these medications do not positively influence the increase in formate in hypertensive patients.

Glycerol is a small organic molecule with three carbons and three hydroxyl groups. It is an important constituent of monoglycerides, diglycerides, and triglycerides, formed by esterification of glycerol and fatty acids. Since the body can store fat as a source of energy, when needed, lipolysis occurs, and glycerol and fatty acids can be released into the blood stream. Then, glycerol can be converted into glucose in liver gluconeogenesis. Regarding glycerol serum levels in our study, we found higher levels in HTo vs. NT, and the presence of comorbidities did not alter these results. Moreover, the AUROC analysis showed a good discriminant power of glycerol for hypertension, independently of the coexistence of other CVMDs. As comorbidities do not have influence, we found also higher levels in HTall vs. NT. The medication with ACEi, ARA, diuretics, BAA, CCB, anticoagulants, or combinations of them, also did not significantly alter serum glycerol levels. Other studies also found an increase in glycerol levels in serum and plasma of hypertensive participants [2,5,18]. Palmu et al. also found that glycerol was one of the strongest predictors of SBP, without any sex-specific or age-specific differences [21]. According to Mahendran et al, glycerol also seems to be a marker of increased risk of developing hyperglycemia and type 2 diabetes [39].

The comparison between NT and HTo showed that hypertensive individuals have lower levels of glutamine, glycine, and sarcosine. Glycine is the simplest non-essential amino acid. Although glycine levels were lower in the HTo group, the levels were significantly higher in the presence of concomitant dyslipidemia (HTdl vs. HTo). On the other hand, the AUROC analysis showed a good discriminant power of glycine for hypertension solely if hypertension does not coexist with other CVMDs. Bai et al. also found a decrease in glycine in human participants with essential hypertension and, since antihypertensive treatment has shown to increase glutathione levels in other studies [40], some authors suggested that it is important to increase glycine levels in participants with essential hypertension [4]. Gil.Redondo et al. also described a decrease in glycine levels related to hypertension and to different metabolic syndrome profiles [37]. In fact, some studies suggested that glycine could have a protective role in hypertension [4,41,42,43]. A study performed by Díaz-Flores et al. analyzed the effects of an oral glycine supplementation in individuals with metabolic syndrome and reported that the male had a significant decrease in SBP (vs. placebo) [43]. Glycine seems to modulate BP through several mechanisms. It was associated with protection of tissues by reduction in oxidative stress (via upregulation of superoxide dismutase), with altering biosynthesis of glutathione (antioxidant preventing damage of cellular components), and with increases in endothelial nitric oxide (induces vasodilation) [4]. Findings from Lin et al. also supported a causal association between higher glycine levels with genetically predicted lower SBP and lower risk of hypertension, and hypothesized that this association could be due to hypertension reducing peripheral glucose utilization, promoting insulin resistance and gluconeogenesis, and accelerating the conversion of glycine to serine [41]. Other studies also showed a negative association between glycine and hypertension [2,18]. Wang et al. hypothesized that this association might be due not only to alteration in generations of free radicals, but also, since glycine is involved in the synthesis of elastin, to a deficient elastin formation [18]. Mels found a negative association between glycine levels and SBP but only in individuals of black ancestry [19]. Other authors demonstrated that the branched-chain amino acid/glycine ratio is a metabolomic signature that is associated with hypertension and coronary heart disease [42]. Glycine has also been inversely associated with cardiovascular diseases such as acute myocardial infarction [44], diabetes [45], and with infarct size in acute ischemic stroke [46]. Medication with ACEi, ARA, diuretics, BAA, CCB, anticoagulants, or combinations did not significantly alter serum glycine levels of hypertensive individuals.

Glutamine is the most abundant amino acid in the human body. It is considered essential depending on the conditions, as in normal conditions, endogenous synthesis is sufficient to meet optimal demands, but it might be insufficient in stress situations or severe illnesses [47]. Glutamine is a substrate for the synthesis of DNA, lipids, proteins, and other non-essential amino acids, and it provides intermediates for the Krebs Cycle to generate ATP [47,48]. It has been associated as a cardiovascular health promoter due to being a citrulline and therefore arginine precursor, leading to an increase in nitric oxide synthesis [47,48]. It also has a strong anti-inflammatory and antioxidant effect because it induces the expression of heme-oxigenase1, heat shock proteins, and glutathione [47]. In our study, glutamine levels were decreased in the HTo group (vs. NT). However, we found significant increases in the subgroup HTcd (vs. HTo), suggesting that the levels increase when hypertension diagnosis is concomitant with cardiac diseases. Moreover, glutamine showed a good discriminant power for hypertension in the AUROC analysis solely if hypertension does not coexist with other CVMD. The medication with ACEi, ARA, diuretics, BAA, CCB, anticoagulants, or combinations did not significantly modify serum glutamine serum levels of hypertensive individuals. Regarding animal studies, Yang et al. exposed a group of Wistar male normotensive rats to a high-salt diet, and then supplemented the rats with different doses of glutamine and found that this supplementation could prevent the development of high-salt-induced hypertension in a dose-dependent manner. High-dose glutamine supplementation significantly lowered SBP and was also inversely associated with left ventricular hypertrophy [48]. Another study performed on mice also compared BP value between mice that were injected intraperitoneally only with saline solution vs. saline solution and glutamine supplement, and found decreased levels of SBP, DBP, and mean arterial pressure in the glutamine-treated group [49]. Yang et al. hypothesized that glutamine, as a precursor of arginine, can lead to an increase in nitric oxide synthesis, therefore decreasing arterial tone [48]. Regarding human studies, Liu et al. used GC-MS in serum samples and found that the glutamine levels were increased in individuals diagnosed with hypertension [30]. However, the study does not mention any exclusion factors related to comorbidities. Goita et al. studied the sexual dimorphism of the metabolomic profile in hypertension in plasma of human adults in Mali (Africa) using LC-MS and found that glutamine was specifically increased in hypertensive women but did not find an increase in men or women and men combined [50]. Another study performed in normotensive people found that black people present significantly increased glutamine levels compared to white people [19]. Therefore, our results in hypertension might differ from these studies because we performed the study in European Caucasian individuals; we differentiated these people, taking into account the comorbidities, and we applied clear exclusion criteria regarding some other conditions or comorbidities.

Sarcosine (N-Methyl-glycine) is an amino acid that is formed in the kidney and liver as an intermediate in the metabolism of choline, by glycine methylation, and it can also be formed by creatine hydrolysis. In this study, we found lower levels of sarcosine in our individuals of the HTo group (vs. NT group). However, when dyslipidemia or cardiac disease was present as comorbidity, the levels increased, which suggests an influence of dyslipidemia in sarcosine levels opposite to that of hypertension. On the other hand, the AUROC analysis showed a good discriminant power of sarcosine for hypertension solely if hypertension does not coexist with other CVMDs. Some authors indicated that elevated sarcosine is a marker of dyslipidemia [37]. Yuan et al. found increased levels of sarcosine in participants with pulmonary arterial hypertension associated with ventricular septal defect [51]. Besides CVMD conditions, sarcosine levels are also elevated in serum and urine from patients with prostate cancer (vs. healthy patients, and vs. patients with benign prostatic hypertrophy), and studies have analyzed its role as a potential prostate cancer biomarker [52,53]. The positive influence of cardiac disease or dyslipidemia, even if does not reach statistical significance, can mitigate the negative influence of hypertension and can be the cause of not having differences in sarcosine levels between non-hypertensive individuals and hypertensive people with other comorbidities (dyslipidemia or cardiac disease). On the other hand, sarcosine levels can be affected by some drugs, as medication with ACEi, AnCoa, CCB, and DIU conjointly, massively, and significantly increase the levels of sarcosine. Other drugs such as ARA or conjointly medication with ARA, anticoagulants, and CCB also increased sarcosine levels (more than 80%) but did not reach statistical significance.

In conclusion, we found higher levels of acetate, formate, and glycerol, and lower levels of glutamine, glycine, and sarcosine related to hypertension, and these six metabolites could be used as biomarkers for hypertension. An increase in formate and glycerol serum levels can be found in all the hypertensive individuals, even with other CVMDs. The increase in acetate occurs in hypertensive individuals but seems to be mitigated by concomitant dyslipidemia and/or diabetes diagnosis and does not have a good discriminant power. A decrease in glutamine occurs in hypertensive individuals but is mitigated if concomitant cardiac diseases exist. A decrease in glycine occurs in hypertensive individuals, but is mitigated by the presence of concomitant dyslipidemia. A decrease in sarcosine occurs in hypertensive individuals but is mitigated by the positive influence of cardiac disease or dyslipidemia if these diseases coexist with hypertension.

## 4. Materials and Methods

### 4.1. Population, Data, and Groups

This study focused on individuals from EBIcohort (Elders from Beira Interior cohort), which were users older than 64 years of eighteen long-term care facilities (LTCF) from three Portuguese municipalities (Fundão, Covilhã, and Belmonte), covering an area of ~1000 square kilometers. The procedures executed in this study were reviewed and approved by the Ethics Committee of the University of Beira Interior in accord with the Helsinki convention (Ref. Number CE-UBI-Pj-2017-012). The participants or their legal representatives signed a written informed before starting the study.

From EBIcohort individuals, we excluded all the participants with at least one of the following conditions: (1) Global Deterioration Scale ≥ 6; (2) diagnosis or history of cancer or tumor malignancy in the previous 5 years; (3) diagnosis of serious psychiatric disorders; (4) use of persistent anticonvulsant therapy; (5) presence of several hematologic disorders with abnormalities in the proportion in blood cells; (6) long treatment with antiretroviral therapy.

Initially, we divided the individuals into two main groups based on the presence or absence of hypertension diagnosis: the control/normotensive group (NT) and the hypertensive group (HTall). Individuals of the NT group did not have any cardiovascular or metabolic diseases (CVMDs), such as acute myocardial infarction, chronic heart failure, angina pectoris, atrial fibrillation, diabetes, or dyslipidemia. The HTall group individuals could have other CVMDs.

To perform further analysis on the influence of other cardiovascular or metabolic diseases (hypertension comorbidities), the HTall group was further subdivided into subgroups based on diagnosis information. Thus, the HTo subgroup is composed of individuals with hypertension but without diagnosis of any other CVMD; HTdl has individuals with only hypertension and dyslipidemia diagnosis as CVDM; HTdb is constituted by individuals with only hypertension and diabetes as CVMD; HTcd with individuals with hypertension and diagnosis of one cardiac disease (either chronic heart failure, angina, acute myocardial infarction, or arrhythmia), but no other CVMD; HTdb•dl formed by individuals with hypertension and further diagnosis of dyslipidemia and diabetes as CVMD; HTcd•dl constituted of individuals with hypertension and diagnosis of dyslipidemia and only one of the abovementioned cardiac diseases as CVMD.

### 4.2. Data and Sample Collection and Processing

Data concerning sociodemographic, clinical, and therapeutic information from each participant were obtained from the individuals and clinical staff of the collaborative LTCF. Body mass index (BMI; kg/m^2^) was calculated using the height and body mass data. Blood pressure (BP) was measured in the same week of the blood and data collection in the LTCF. All the measurements were performed in standard conditions, using a manual or automated sphygmomanometer, the gold standard method to evaluate BP. Systolic blood pressure (SBP) and diastolic blood pressure (DBP) results are expressed in mmHg.

The cognitive status of the participants was assessed by a competent team from our group through the application of the Global Deterioration Scale (GDS) and the Addenbrooke’s Cognitive Examination-Revised (ACE-R) test. The GDS is a scale that was developed to evaluate primary degenerative dementia, measuring the cognitive decline over time in seven different progressing stages, considering clinical characteristics and memory deterioration of the patients [54]. The ACE-R is a rapid test battery, with good sensitivity and specificity, to screen for early cognitive impairment and dementia, and it can be used to distinguish between frontotemporal dementia and Alzheimer’s Disease [55]. It evaluates six cognitive domains: attention/orientation, memory, fluency, language, and visuospatial ability; two of them are part of the Mini-Mental State Examination (MMSE) [55]. This test was already validated for the Portuguese population [56]. MMSE is shorter and provides rapid screening of cognitive state: distinguishing between normal cognition and mild, moderate, and severe cognitive impairment.

Fasting serum samples were collected from all the individuals within the same week of data collection, cognitive evaluation, and blood pressure measurement. Venous blood samples were collected into serum-collecting tubes after overnight fasting of individuals, then were left at room temperature to complete the clotting process (10–20 min). Samples were then centrifuged at 1500× *g* for 10 min at room temperature. Still within 10–15 min after centrifugation, four serum aliquots from each participant (400 µL) were stored at −20 °C and, ~3 h after, were cryopreserved (−80 °C freezer) until further NMR processing.

We measured several biochemical analytes in all serum samples, namely glucose, triglycerides (TG), total cholesterol, high-density lipoprotein (HDL) cholesterol, using spectrophotometric colorimetric methods. All reagents were bought from BioSystems Inc. (Atlanta, GA, USA). We determined low-density lipoprotein (LDL) cholesterol levels by using the Friedewald equation.

### 4.3. NMR Experiment, Analysis, and Quantification

Frozen serum samples were thawed at room temperature and were manually prepared. Each serum sample was mixed with serum buffer (75 mM Na_2_HPO_4_, 2 mM NaN_3_, 4.6 mM sodium trimethylsilyl propionate-[2,2,3,3-2H_4_] (TSP) in 10% D_2_O, pH 7.4 ± 0.1) in a 1:1 (*v*/*v*) ratio for a final volume of 600 µL into the 5 mm NMR tube.

NMR measurements were conducted in a 600 MHz IVDr (Bruker BioSpin, Silberstreifen, Germany) with a tempered SampleJet automatic sample changer and a double resonance broadband probe (BBI) probe head with a z gradient coil and BOSS-III shim system. NMR sample tubes were stored inside the SampleJet at 5 °C until measurement.

Every morning, the spectrometer was calibrated with three different samples: methanol, QuantRef, and sucrose to check the temperature (310 K), the quantification performance, and optimal shimming, respectively, following strict standard operation procedures, as previously described [57].

Several different 1H NMR experiments were recorded in all the samples: a one-dimensional (1D) NOESY (Nuclear Overhauser Effect Spectroscopy) 32-scan NMR experiment was used to show the NMR spectrum quality (via the B.I. BioBankQC^TM^, Tokyo, Japan) and to enable the quantification of metabolites of the B.I. BioBankQuant-PS^TM^. Then, a 1D 32-scan CPMG (Carr–Purcell–Meiboom–Gill, filtering out macromolecular resonance signals) program was run. Moreover, a two-dimensional (2D) 2-scan JRES (J-RESolved spectroscopy), was included with the IVDr methods to analyze J coupling constants. Also, for samples with unknown peaks, we also included a 2D TOCSY (Total Correlation Spectroscopy) and a 2D HSQC (Heteronuclear Single Quantum Coherence) to identify the unknown metabolites.

**Figure 7:**
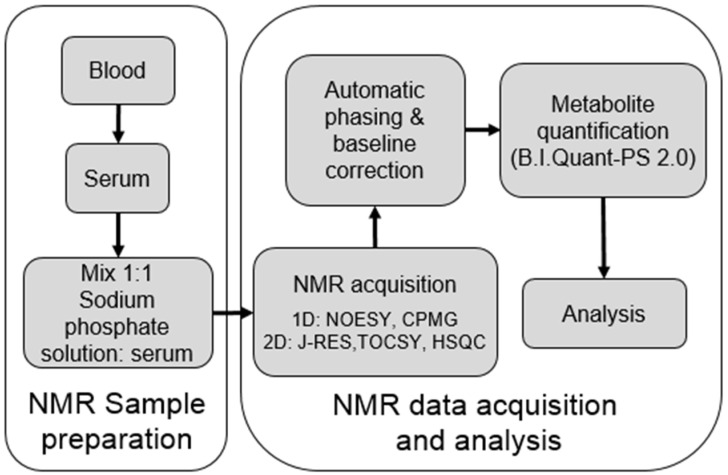
NMR analysis flowchart for human serum metabolomic studies.

Absolute quantifications from 1H-NMR spectra were performed with Bruker IVDr software B.I.Quant-PS 2.0.0 to quantify 37 serum metabolites (mmol/L units): 2-Aminobutyrate, 2-Hydroxybutyrate, 2-Oxoglutate, 3-Hydroxybutyrate, Acetate, Acetoacetate, Acetone, Alanine, Asparagine, Choline, Citrate, Creatine, Creatinine, D-Galactose, Ethanol, Formate, Glucose, Glutamate, Glutamine, Glycerol, Glycine, Isoleucine, Lactate, Leucine, Lysine, Methionine, N,N-Dimethylglycine, Ornithine, Phenylalanine Proline, Pyruvate, Sarcosine, Succinate, Threonine, Trimethylamine-N-oxide, Tyrosine, Valine. A flowchart of the NMR analysis procedure is shown in Figure 7. The NMR limit of detection of these metabolites is in Table S6.

### 4.4. Statistical Analysis

NMR data were managed using SPSS version 28, and the analysis was performed using Student’s *t*-test (TTS), Mann–Whitney test (MWT), One-way ANOVA test (OWAT), Kruskal–Wallis test (KWT), and Fisher’s exact test (FET). *p* Values under 0.05 were considered statistically significant.

## Figures and Tables

**Table 1:** Characterization and comparison of sociodemographic, BP, biochemical, and cognitive data of NT vs. HTall groups and NT vs. HTo groups. Data are expressed as “mean ± s.e.m.” except for sex, expressed as “percent (n)”. Statistical analysis was performed using Student’s *t*-test (STT), except for sex, where Fisher’s exact test (FET) was used.

PARAMETERS	NT	HTall	*p* Value	HTo	*p* Value
SOCIODEMOGRAPHIC DATA					
Number (n)	11	115		22	
Age (years)	77.82 ± 3.45	84.23 ± 0.69	0.048	81.32 ± 2.15	0.376
Sex, female (% (n))	45.50 (5)	71.30 (82)	0.080	54.50 (12)	0.645
Body mass index (kg/m^2^)	25.09 ± 1.51	27.58 ± 0.51	0.057	27.29 ± 1.76	0.394
BLOOD PRESSURE					
Systolic blood pressure (mmHg)	125.64 ± 3.14	126.32 ± 2.10	0.429	126.32 ± 4.27	0.898
Diastolic blood pressure (mmHg)	69.73 ± 3.71	69.08 ± 0.99	0.423	72.43 ± 2.12	0.501
BIOCHEMICAL TEST RESULTS					
Serum glucose (mg/dL)	94.65 ± 5.12	99.20 ± 2.62	0.298	94.32 ± 2.72	0.950
Serum triglycerides (mg/dL)	112.03 ± 12.15	115.64 ± 5.38	0.419	108.27 ± 2.02	0.821
Serum total cholesterol (mg/dL)	187.63 ± 8.65	164.74 ± 4.58	0.064	190.37 ± 8.96	0.845
Serum HDL cholesterol (mg/dL)	59.56 ± 3.96	54.51 ± 1.15	0.099	55.32 ± 2.39	0.340
Serum LDL cholesterol (mg/dL)	105.67 ± 7.05	87.11 ± 3.98	0.078	113.39 ± 9.34	0.584
COGNITIVE TESTS RESULTS					
Revised Addenbrooke’s Cognitive Test (ACE-R)	48.20 ± 5.34	48.22 ± 2.00	0.998	46.70 ± 4.75	0.847
Mini-Mental State Examination (MMSE)	19.45 ± 1.42	17.60 ± 0.58	0.322	17.10 ± 1.58	0.332
Global Deterioration Scale (GDS)	3.18 ± 0.33	3.11 ± 0.15	0.877	3.24 ± 0.34	0.917

**Table 2:** Characterization and comparison of the main drugs used in hypertension vs. HTall groups and NT vs. HTo groups. Data are expressed as “percent (n)”. Statistical correlation analysis was performed using Fisher’s exact test (FET). * *p* < 0.05 when compared with NT. “-” means that the calculation is not applicable because in one of the groups compared n = 0. We can substitute bi N.A. or NA (not applicable).

TREATMENTS	NT	HTall	*p* Value NT vs. HTall	HTo	*p* Value NT vs. HTo
Number (n)	11	115		22	
Antiarrhythmics	0.0 (0)	4.3 (5)	0.628	0.0 (0)	-
Antianginal	0.0 (0)	14.8 (17)	0.189	4.5 (1)	0.667
ACEi	0.0 (0)	23.5 (27)	0.062	27.3 (6)	0.067
ARA	0.0 (0)	53.0 (61)	<0.001 *	45.5 (10)	0.007 *
BAA	0.0 (0)	20.9 (24)	0.088	13.6 (3)	0.282
CCB	0.0 (0)	26.1 (30)	0.043 *	13.6 (3)	0.282
Diuretics	0.0 (0)	69.6 (80)	<0.001 *	50.0 (11)	0.004 *
Venotropics	18.2 (2)	15.7 (18)	0.550	13.6 (3)	0.550
Anticoagulants	18.2 (2)	59.1 (68)	0.010 *	54.5 (12)	0.051
Sulfonylureas	0.0 (0)	4.3 (5)	0.628	0.0 (0)	-
Biguanides	0.0 (0)	17.4 (20)	0.137	0.0 (0)	-
DPP-4 inhibitors	0.0 (0)	17.4 (20)	0.137	0.0 (0)	-
Insulin	0.0 (0)	7.8 (9)	0.427	0.0 (0)	-
Statins	18.2 (2)	46.1 (53)	0.068	0.0 (0)	-
Bronchodilators	0.0 (0)	17.4 (20)	0.137	0.0 (0)	-
AChE inhibitors	0.0 (0)	8.7 (10)	0.598	13.6 (3)	0.282
MAO inhibitors	0.0 (0)	0.9 (1)	1.000	0.0 (0)	-
NMDA antagonist	0.0 (0)	8.7 (10)	0.598	4.5 (1)	0.667
Antiepileptics	18.2 (2)	4.3 (5)	0.114	4.5 (1)	0.252
Antipsychotics	36.4 (4)	27.8 (32)	0.509	36.4 (8)	0.645
Antidepressants	27.3 (3)	45.2 (52)	0.346	40.9 (9)	0.355

**Table 3:** Changes in proportion of acetate, acetoacetate, formate, and glucose levels between the HTall group of individuals without therapy of ACEi, ARA, DIU, BAA, CCB, or anticoagulants (AnCoa) and individuals with therapies containing ACEi. ▼ = decrease; ▲ = increase. Data are expressed as “percent of change” compared with individuals not medicated with ACEi, ARA, DIU, BAA, CCB, or anticoagulants therapies.

THERAPIES (n)	Mean ± s.e.m.	Percent of Change	Mean ± s.e.m.	Percent of Change
	ACETATE		ACETOACETATE	
Without drugs (4)	0.0295 ± 0.0063		0.0195 ± 0.0100	
ACEi (3)	0.0380 ± 0.0120	▲28.8%	0.0120 ± 0.0031	▼38.5%
ACEi + AnCoa (4)	0.0155 ± 0.0045	▼47.5%	0.0110 ± 0.0052	▼43.6%
ACEi + AnCoa + DIU (7)	0.0249 ± 0.0040	▼15.6%	0.0269 ± 0.0202	▲37.9%
ACEi + AnCoa + CCB + DIU (5)	0.0342 ± 0.0068	▲15.9%	0.0148 ± 0.0074	▼24.1%
ARA (8)	0.0205 ± 0.0033	▼30.5%	0.0184 ± 0.0106	▼5.6%
ARA + DIU (13)	0.0257 ± 0.0044	▼12.9%	0.0140 ± 0.0038	▼28.2%
ARA + AnCoa + DIU (13)	0.0295 ± 0.0057	0%	0.0173 ± 0.0045	▼11.3%
ARA + AnCoa + CCB (4)	0.0323 ± 0.0112	▲9.5%	0.0150 ± 0.0061	▼23.1%
ARA + AnCoa + DIU + CCB (7)	0.0243 ± 0.0041	▼17.6%	0.0209 ± 0.0061	▲7.2%
DIU (6)	0.0275 ± 0.0072	▼6.8%	0.0068 ± 0.0021	▼65.1%
DIU + AnCoa (5)	0.0344 ± 0.0103	▲16.6%	0.0152 ± 0.0045	▼22.1%
DIU + AnCoa +BAA (4)	0.0245 ± 0.0032	▼16.9%	0.0150 ± 0.0044	▼23.1%
	FORMATE		GLUCOSE	
Without drugs	0.0373 ± 0.0071		5.311 ± 0.5468	
ACEi	0.0323 ± 0.0034	▼13.4%	5.1093 ± 0.7319	▼3.8%
ACEi + AnCoa	0.0258 ± 0.0030	▼30.8%	5.0998 ± 0.4259	▼4.0%
ACEi + AnCoa + DIU	0.0329 ± 0.0019	▼11.8%	5.2799 ± 0.3706	▼0.6%
ACEi + AnCoa + CCB + DIU	0.0319 ± 0.0018	▼14.5%	5.6760 ± 0.6433	▲6.9%
ARA	0.0305 ± 0.0030	▼18.2%	4.9129 ± 0.1766	▼7.5%
ARA + DIU	0.0305 ± 0.0015	▼18.2%	5.6579 ± 0.4689	▲6.5%
ARA + AnCoa + DIU	0.0305 ± 0.0018	▼18.2%	6.1078 ± 0.3766	▲15.0%
ARA + AnCoa + CCB	0.0290 ± 0.0030	▼22.3%	7.0460 ± 1.4553	▲32.7%
ARA + AnCoa + DIU + CCB	0.0320 ± 0.0031	▼14.2%	7.4503 ± 1.4988	▲40.3%
DIU	0.0332 ± 0.0041	▼11.0%	5.9345 ± 0.4818	▲11.7%
DIU + AnCoa	0.0358 ± 0.0048	▼4.0%	5.7498 ± 0.8535	▲8.3%
DIU + AnCoa +BAA	0.0275 ± 0.0026	▼26.3%	5.331 ± 0.7177	▲0.4%

**Table 4:** HTall changes in proportion of glutamine, glycerol, glycine, and sarcosine levels between groups of individuals without therapy of ACEi, ARA, DIU, BAA, CCB, or anticoagulants (AnCoa) and individuals with different therapies with these drug classes. ▼ = decrease; ▲ = increase. Data are expressed as “percent of change” compared with individuals without ACEi, ARA, DIU, BAA, CCB, or anticoagulants therapies. * The comparison is level of the metabolite without drugs.

THERAPIES	Mean ± s.e.m.	Percent of Change	Mean ± s.e.m.	Percent of Change
	GLUTAMINE		GLYCEROL	
Without drugs	0.7973 ± 0.0315		0.2273 ± 0.0904	
ACEi	0.7660 ± 0.1131	▼3.9%	0.2147 ± 0.0618	▼5.5%
ACEi + AnCoa	0.7408 ± 0.0308	▼7.1%	0.2378 ± 0.1149	▲4.6%
ACEi + AnCoa + DIU	0.8200 ± 0.0484	▲2.8%	0.2577 ± 0.0706	▲13.4%
ACEi + AnCoa + CCB + DIU	0.7490 ± 0.0333	▼6.1%	0.1120 ± 0.0713	▼50.7%
ARA	0.7973 ± 0.0315	▲9.8%	0.2090 ± 0.0503	▼8.1%
ARA + DIU	0.8758 ± 0.0444	▼3.2%	0.3042 ± 0.0361	▲33.8%
ARA + AnCoa + DIU	0.7717 ± 0.024	▼0.4%	0.2142 ± 0.0451	▼5.8%
ARA + AnCoa + CCB	0.7945 ± 0.0349	▼3.2%	0.1223 ± 0.0727	▼46.2%
ARA + AnCoa + DIU + CCB	0.7720 ± 0.0232	▼4.7%	0.3059 ± 0.0806	▲34.6%
DIU	0.8030 ± 0.0257	▲0.7%	0.1487 ± 0.0722	▼34.6%
DIU + AnCoa	0.8162 ± 0.0826	▲2.4%	0.2228 ± 0.1068	▼2.0%
DIU + AnCoa +BAA	0.7168 ± 0.0281	▼10.1%	0.3155 ± 0.0582	▲38.8%
	GLYCINE		SARCOSINE	
Without drugs	0.271 ± 0.0276		0.0040 ± 0.0011	
ACEi	0.284 ± 0.034	▲4.8%	0.0037 ± 0.0009	▼7.5%
ACEi + AnCoa	0.2468 ± 0.0423	▼8.9%	0.0063 ± 0.002	▲57.5%
ACEi + AnCoa + DIU	0.3049 ± 0.0139	▲12.5%	0.0056 ± 0.0018	▲40.0%
ACEi + AnCoa + CCB + DIU	0.2668 ± 0.0713	▼1.5%	0.0098 ± 0.0024	▲145.0% *
ARA	0.3236 ± 0.0388	▲19.4%	0.0073 ± 0.0023	▲82.5%
ARA + DIU	0.2725 ± 0.0123	▲0.6%	0.0049 ± 0.001	▲22.5%
ARA + AnCoa + DIU	0.2502 ± 0.0106	▼7.7%	0.0036 ± 0.001	▼10.0%
ARA + AnCoa + CCB	0.2505 ± 0.0298	▼7.6%	0.0095 ± 0.0029	▲137.5%
ARA + AnCoa + DIU + CCB	0.2443 ± 0.0208	▼9.9%	0.0056 ± 0.0011	▲40.0%
DIU	0.2818 ± 0.0326	▲4.0%	0.0032 ± 0.0012	▼20.0%
DIU + AnCoa	0.3048 ± 0.0488	▲12.5%	0.0068 ± 0.0047	▲70.0%
DIU + AnCoa +BAA	0.3238 ± 0.0579	▲19.5%	0.0063 ± 0.0015	▲57.5%

## Data Availability

The data that support the findings of this study are available on request from the corresponding author, I. Verde. The data are not publicly available due to restrictions of data protection law (Portugal) because they contain information that could compromise the privacy of research participants.

## Supplementary Materials

The following supporting information can be downloaded at: https://www.mdpi.com/article/10.3390/molecules30102145/s1.

## Abbreviations

The following abbreviations are used in this manuscript:

ACEi	Angiotensin-Converting Enzyme inhibitors
ARA	Angiotensin-Receptor Antagonists
BAA	Beta-Adrenergic Antagonists
BP	Blood Pressure
CCB	Calcium Channel Blockers
WHO	World Health Organization
