# Supplementary material for: Nuclear Magnetic Resonance Analysis Seeking for Metabolic Markers of Hypertension in Human Serum
Source: Molecules. 2025 May 13;30(10):2145. doi: 10.3390/molecules30102145 (PMC12113710; doi:10.3390/molecules30102145)
Supplement: Supplementary file 1 [file molecules-30-02145-s001.zip › molecules-3592484-supplementary.pdf]

Table S1 Relative metabolite serum concentration of metabolites and comparison between NT and HTo groups. Data are expressed as "mean ± s.e.m". Statistical significance between groups was analyzed using Student t-test (STT).

	Control	HTo	P
Acetate	0.022±0.002	0.032±0.004	0.025
Acetoacetate	0.01±0.003	0.019±0.004	0.124
Acetone	0.021±0.003	0.02±0.002	0.791
Alanine	0.46±0.039	0.446±0.02	0.733
Alpha-aminobutyrate	0.038±0.008	0.029±0.006	0.398
Alpha-hydroxybutyrate	0.018±0.008	0.016±0.008	0.879
Alpha-ketoglutarate	0.004±0.002	0.006±0.001	0.416
Asparagine	0.046±0.01	0.04±0.006	0.548
Beta-hydroxybutyrate	0.055±0.016	0.057±0.01	0.886
Choline	0.003±0.001	0.005±0.002	0.446
Citrate	0.179±0.01	0.179±0.008	0.99
Creatine	0.025±0.006	0.027±0.004	0.765
Creatinine	0.095±0.005	0.105±0.009	0.462
Dimethylsulfone	0.011±0.002	0.013±0.002	0.460
Ethanol	0.016±0.007	0.019±0.005	0.768
Formate	0.025±0.001	0.034±0.001	<.001
Glucose	5.258±0.267	5.176±0.198	0.810
Glutamate	0.039±0.008	0.056±0.007	0.141
Glutamine	0.839±0.040	0.744±0.023	0.037
Glycerol	0.107±0.040	0.227±0.030	0.027
Glycine	0.298±0.018	0.255±0.011	0.036
Histidine	0.088±0.005	0.082±0.003	0.252
Isoleucine	0.05±0.004	0.054±0.004	0.516
Lactate	2.198±0.188	2.244±0.164	0.863
Leucine	0.092±0.005	0.083±0.006	0.341
Lysine	0.178±0.027	0.211±0.017	0.289
Methionine	0.077±0.005	0.077±0.004	0.989
N,N-Dimethylglycine	0.006±0	0.007±0.001	0.613
Ornithine	0.054±0.012	0.04±0.008	0.372
Phenylalanine	0.059±0.004	0.058±0.002	0.694
Proline	0.188±0.047	0.241±0.038	0.414
Pyruvate	0.04±0.010	0.064±0.01	0.140
Sarcosine	0.007±0.001	0.004±0.001	0.023
Succinate	0.004±0.001	0.004±0	0.277
Threonine	0.191±0.018	0.169±0.02	0.474
Trimethylamine N-Oxide	0.041±0.007	0.038±0.005	0.743
Tyrosine	0.066±0.004	0.065±0.004	0.841
Valine	0.228±0.011	0.229±0.014	0.959

Table S2. Relative metabolite serum concentration of metabolites and comparison between NT and HTall groups. Data are expressed as "mean ± s.e.m". Statistical significance between groups was analyzed using Student t-test (STT).

	NT	HTall	P (STT)
Acetate	0.022±0.002	0.025±0.001	0.227
Acetoacetate	0.010±0.003	0.015±0.002	0.378
Acetone	0.021±0.003	0.022±0.001	0.721
Alanine	0.46±0.039	0.465±0.011	0.883
Alpha-aminobutyrate	0.038±0.008	0.028±0.002	0.226
Alpha-hydroxybutyrate	0.018±0.008	0.017±0.003	0.883
Alpha-ketoglutarate	0.004±0.002	0.007±0.001	0.062
Asparagine	0.046±0.01	0.038±0.003	0.386
Beta-Hydroxybutyrate	0.055±0.016	0.067±0.006	0.508
Choline	0.003±0.001	0.006±0.001	0.044
Citrate	0.179±0.01	0.191±0.004	0.374
Creatine	0.025±0.006	0.031±0.002	0.464
Creatinine	0.095±0.005	0.115±0.004	0.004
Dimethylsulfone	0.011±0.002	0.021±0.004	0.449
Ethanol	0.016±0.007	0.017±0.003	0.847
Formate	0.025±0.001	0.031±0.001	<0.001
Glucose	5.258±0.267	5.765±0.178	0.385
Glutamate	0.039±0.008	0.066±0.004	0.036
Glutamine	0.839±0.04	0.784±0.011	0.148
Glycerol	0.107±0.04	0.245±0.015	0.007
Glycine	0.298±0.018	0.276±0.006	0.289
Histidine	0.088±0.005	0.084±0.001	0.301
Isoleucine	0.05±0.004	0.056±0.002	0.254
Lactate	2.198±0.188	2.095±0.052	0.561
Leucine	0.092±0.005	0.089±0.002	0.707
Lysine	0.178±0.027	0.192±0.008	0.634
Methionine	0.077±0.005	0.075±0.002	0.772
N.N-Dimethylglycine	0.006±0	0.007±0.000	0.498
Ornithine	0.054±0.012	0.048±0.004	0.709
Phenylalanine	0.059±0.004	0.059±0.001	0.972
Proline	0.188±0.047	0.25±0.018	0.296
Pyruvate	0.04±0.01	0.054±0.003	0.180
Sarcosine	0.007±0.001	0.006±0.000	0.318
Succinate	0.004±0.001	0.004±0.000	0.751
Threonine	0.191±0.018	0.171±0.009	0.496
Trimethylamine N-Oxide	0.041±0.007	0.042±0.003	0.891
Tyrosine	0.066±0.004	0.062±0.002	0.507
Valine	0.228±0.011	0.233±0.005	0.794

Table S3. *Relative mean serum concentration of metabolites that showed significant differences between HTo and subgroups of individuals diagnosed with dyslipidemia (HTdl), dyslipidemia plus one cardiac disease (HTcd•dl) and dyslipidemia plus one cardiac disease (HTcd•dl). Data are expressed as "mean ± s.e.m". Statistical significance was analyzed using Kruskal-Wallis test (KWT) or One-way ANOVA test (OWAT) depending on the homogeneity of variance (P value close to metabolite names). Differences among subgroups were analyzed by using post hoc Tukey test or all pairwise nonparametric method (*P < 0.05 compared with HTo; # P < 0.05 compared with HTdb•dl).*

	HTo	HTdl	HTdb•dl	HTcd•dl
Acetate (P=0.008)	0.032±0.004	0.022±0.002	0.016±0.002*	0.021±0.003
Formate (P=0.041)	0.034±0.001	0.031±0.001	0.032±0.002	0.028±0.002*
Glycine (P=0.009)	0.256±0.011	0.296±0.012*	0.259±0.012	0.334±0.024*#
Sarcosine (P=0.013)	0.004±0.001	0.007±0.001	0.007±0.001	0.009±0.002*

Table S4. *Relative mean serum concentration of metabolites that showed significant differences between HTo and groups with individuals diagnosed with diabetes (HTdb). dyslipidemia plus diabetes (HTdb•dl) and one cardiac disease plus diabetes (HTcd•db). Data are expressed as "mean ± s.e.m". Statistical significance was analyzed using Kruskal-Wallis test (KWT) or One-way ANOVA test (OWAT) depending on the homogeneity of variance (P value close to metabolite names). Differences among subgroups were analyzed by using post hoc Tukey test or all pairwise nonparametric method (*P < 0.05 compared with HTo; # P < 0.05 compared with HTdb•dl).*

	HTo	HTdb	HTdb•dl	HTcd•db
Acetate (P=0.034)	0.032±0.004	0.023±0.002	0.016±0.002*	0.016±0.002*
Acetoacetate (P=0.045)	0.019±0.004	0.009±0.003	0.005±0.001*	0.019±0.006#
Glucose (P=0.025)	5.176±0.198	6.891±1.026	6.920±0.816	7.847±1.173*
Glycerol (P=0.045)	0.227±0.030	0.191±0.055	0.181±0.067	0.388±0.031

Table S5. *Relative mean serum concentration of metabolites that showed significant differences between HTo and subgroups of individuals diagnosed with cardiac disease (HTcd). dyslipidemia plus one cardiac disease (HTcd•dl) and diabetes plus one cardiac disease (HTcd•db). Data are expressed as "mean ± s.e.m". Statistical significance was analyzed using Kruskal-Wallis test (KWT) or One-way ANOVA test (OWAT) depending on the homogeneity of variance (P value close to metabolite names). Differences among subgroups were analyzed by using post hoc Tukey test or all pairwise nonparametric method (*P < 0.05 compared with HTo; & P < 0.05 compared with HTcd; # P < 0.05 compared with HTcd•dl).*

	HTo	HTcd	HTcd•dl	HTcd•db
Acetate (P=0.034)	0.032±0.004	0.029±0.007	0.021±0.003	0.016±0.002*&
Formate (P=0.012)	0.034±0.001	0.027±0.003	0.028±0.002	0.027±0.002
Glucose (P=0.017)	5.176±0.198	5.418±0.266	5.174±0.378	7.847±1.173*
Glutamine (P=0.003)	0.745±0.023	0.884±0.032*	0.778±0.022	0.710±0.039&
Glycerol (P=0.040)	0.227±0.030	0.225±0.048	0.205±0.042	0.388±0.031*#
Glycine (P=0.005)	0.256±0.011	0.291±0.025	0.334±0.024*	0.240±0.020#
Sarcosine (P=0.022)	0.004±0.001	0.006±0.002	0.009±0.002*	0.004±0.001

Table S6. *Limit of Detection of metabolites using NMR measurements were done in a 600 MHz IVDr (Bruker BioSpin, Silberstreifen, Germany). From "Analysis Report Bruker IVDr Quantifiation in Plasma/Serum B.I.Quant-PS^{TM}", 2025 (Bruker Spin Company).*

	**LOD (mM)**
Acetate	0.01
Acetoacetate	0.01
Acetone	0.02
Alanine	0.02
Alpha-aminobutyrate	0.05
Alpha-hydroxybutyrate	0.15
Alpha-ketoglutarate	0.02
Asparagine	0.05
Beta-Hydroxybutyrate	0.02
Choline	0.05
Citrate	0.03
Creatine	0.01
Creatinine	0.01
Dimethylsulfone	0.01
Ethanol	0.10
Formate	0.02
Glucose	0.54
Glutamate	0.05
Glutamine	0.02
Glycerol	0.08
Glycine	0.01
Histidine	0.02
Isoleucine	0.03
Lactate	0.03
Leucine	0.01
Lysine	0.04
Methionine	0.05
N,N-Dimethylglycine	0.01
Ornithine	0.02
Phenylalanine	0.03
Proline	0.05
Pyruvate	0.03
Sarcosine	0.01
Succinate	0.01
Threonine	0.04
Trimethylamine N-Oxide	0.08
Tyrosine	0.03
Valine	0.03
